# Self-Evaluative and Other-Directed Emotional and Behavioral Responses to Gossip About the Self

**DOI:** 10.3389/fpsyg.2018.02603

**Published:** 2019-01-04

**Authors:** Elena Martinescu, Onne Janssen, Bernard A. Nijstad

**Affiliations:** ^1^King's Business School, King's College London, London, United Kingdom; ^2^Department of Human Resource Management & Organizational Behaviour, University of Groningen, Groningen, Netherlands

**Keywords:** gossip, self, emotions, prosocial behavior, antisocial behavior, core self evaluations, concern for reputation, feedback

## Abstract

Gossip, or informal talk about others who are not present, is omnipresent in daily interactions. As such, people who are targeted are likely to hear some gossip about themselves, which may have profound implications for their well-being. We investigated the emotions and behavioral intentions of people who hear performance-related gossip about themselves. Based on the affective events theory, we predicted that gossip incidents have strong emotional consequences for their targets and that these emotional responses trigger different behaviors. Two scenario studies (*N*_1_ = 226, *M*_age_ = 21.76; *N*_2_ = 204, *M*_age_ = 34.11) and a critical incident study (*N* = 240, *M*_age_ = 37.04) compared targets' responses to positive and negative gossip. Whereas, targets of positive gossip experienced positive self-conscious emotions (e.g., pride), targets of negative gossip experienced negative self-conscious emotions (e.g., guilt), especially when they had low core self-evaluations. In turn, these negative self-conscious emotions predicted repair intentions. Positive gossip also led to positive other-directed emotions (e.g., liking), which predicted intentions to affiliate with the gossiper. Negative gossip, however, also generated other-directed negative emotions (e.g., anger), especially for targets with high reputational concerns, which in turn predicted retaliation intentions against the gossiper. This pattern of emotional reactions to self-relevant gossip was found to be unique and different from emotional reactions to self-relevant feedback. These results show that gossip has self-evaluative and other-directed emotional consequences, which predict how people intend to behaviorally react after hearing gossip about themselves.

## Introduction

People talk about others abundantly and such conversations about others play a pivotal role in the informal communication network in the workplace (Noon and Delbridge, 1993). Research suggests that up to 70% of our daily conversations contain positive or negative informal evaluations about someone who is not present (Dunbar et al., 1997), a type of interpersonal communication known as gossip (Foster, 2004). Accordingly, gossip has been portrayed as intrinsic to human nature and essential for group functioning (Dunbar, 2004). For example, the threat of becoming a gossip target has been found to deter self-serving behavior and to increase group-serving behavior in social dilemma situations (e.g., Beersma and van Kleef, 2011; Feinberg et al., 2012).

Although gossip is typically not intended to be heard by the *gossip targets* (i.e., people whom the gossip is about; Foster, 2004), gossip does occasionally leak out of the closed gossip circle and reaches its targets. As gossip is evaluative in nature and can damage or boost people's self-evaluation and reputation (Burt, 2008), it may have profound implications for its targets' well-being and is likely to induce specific emotions and behaviors. Negative gossip represents a social judgment about undesirable behaviors or characteristics, which may reduce target's self-confidence, as well as others' trust and willingness to cooperate with the target (e.g., Sommerfeld et al., 2007; Beersma and van Kleef, 2011). In contrast, positive gossip may benefit the target, because it promotes a positive view of the self and signals support and social inclusion (Merry, 1984; Burt, 2008; Ellwardt et al., 2012). Despite these profound consequences, empirical research has not yet clarified how people react when they do hear gossip about themselves. Thus, although we know that the *threat* of gossip may increase pro-group behavior, we do not know the consequences of actually *being* a gossip target.

In this contribution, we used the affective events theory (AET, Weiss and Cropanzano, 1996) to examine how gossip targets feel and behave. Based on the AET, we predicted that gossip targets experience complex emotional and behavioral responses, that are shaped by the valence of the gossip incident (positive vs. negative) and its implications for self-evaluation and reputation. Specifically, we propose that gossip is an affective event, and employees who are confronted with negative gossip about themselves manifest the intention to repair their mistakes, due to self-conscious negative emotions (e.g., guilt), but also the intention to retaliate against the gossiper, due to other-directed negative emotions (e.g., anger). Thus, becoming the target of especially negative gossip may, on the one hand, stimulate group-serving behavior in the form of repair intentions, but it may also trigger more destructive behaviors such as retaliation. Those who encounter positive gossip about themselves might experience self-conscious positive emotions (e.g., pride) and/or other-directed positive emotions (e.g., liking), which may increase the development of affiliation intentions.

We examined two further issues. First, we investigated whether the predicted patterns of event-emotion-behavior (cf. AET, Weiss and Cropanzano, 1996) are contingent on the type of information people receive about themselves. Although (informal) gossip about the self and (formal) feedback may convey the same message about someone's behavior or abilities, they have a different intended audience and purpose: feedback is directed at the target person and meant to help the target, whereas gossip is not. Thus, we propose that being the target of gossip induces emotional reactions that are unique and different from the emotional reactions employees experience when they receive evaluative feedback about the self. Second, we investigated whether the emotions and behaviors individuals experience in response to gossip are contingent on their predispositions. We identified two dispositional traits that moderate the effect of gossip valence on emotions and behavioral intentions: core self-evaluations and concern for reputation. Because being the target of gossip may arouse both self-conscious and other-directed emotions, and because the behavioral consequences of those distinct emotions are quite different, it is important to understand when self-conscious vs. other-directed emotional responses are more likely.

## Theoretical Framework

Because maintaining positive evaluations about the self and a positive reputation among group members are fundamental to well-being (Kunda, 1990; Sedikides and Strube, 1997; Anseel et al., 2007), people are emotionally sensitive to hearing evaluations about their attributes or performance (Frijda, 1988). As such, hearing gossip about the self is likely to cause strong affective responses and behavioral manifestations. Indeed, according to Weiss and Cropanzano's (1996) affective events theory (AET), incidents at work, such as gossip incidents, are likely to trigger emotional reactions. As a theory of workplace emotion, the AET offers a framework for explaining attitudes and behavior in the workplace, by incorporating within-person changes in affective states (Weiss and Beal, 2005; Cropanzano et al., 2017). Specifically, the AET conceptualizes events in the workplace as the cause of affective reactions, which have a direct influence on affect-based behaviors. Furthermore, the AET considers the role of individual predispositions in shaping behavior, by influencing affective reactions to events.

Consistent with the AET, we propose that receiving gossip about the self represents an affective event that generates complex affective experiences and behavioral manifestations. During critical events (such as hearing gossip about the self), emotions prioritize psychological experiences and behaviors, helping individuals respond to environmental challenges or opportunities and adjust their goals (Oatley and Johnson-Laird, 1987). Furthermore, emotions trigger certain behavioral responses (or behavioral intentions) that are aimed at achieving these goals (e.g., Frijda, 1988).

### Self-Conscious Emotional Reactions and Behavioral Intentions

Gossip provides targets with information about their performance, abilities, moral behavior, or other self-relevant issues (Peters and Kashima, 2014), which may support or discredit their positive self-image (Argyle, 1969; Kunda, 1990; Sedikides and Strube, 1997). At face value, gossip about the self represents impromptu positive or negative feedback, which targets can use to evaluate their past behavior or their attributes and act more in line with the pressures and demands of their environment. Drawing from the AET, because gossip contains evaluative information about the self, it is a meaningful self-relevant event that is likely to evoke self-conscious emotional reactions and subsequent emotion-based behavior.

Self-conscious emotions arise when individuals examine their own traits or behavior and are pleased or displeased with themselves (Tracy and Robins, 2004). In the self-evaluation process, individuals compare characteristics of the self with certain social, normative standards, which helps them maintain their place in the social hierarchy. Because gossipers analyze targets' behavior in relation to social norms, gossip is, by definition, normative (e.g., Beersma and Van Kleef, 2012; Feinberg et al., 2012; Peters and Kashima, 2014) and is therefore likely to evoke self-conscious emotions for targets.

Negative gossip raises targets' awareness of their inadequate attributes or behavior (e.g., substandard performance, task failure, avoiding responsibilities). Targets of negative gossip may feel responsible for not meeting certain standards or for harming or disappointing others and are likely to experience self-conscious negative emotions, such as guilt and shame (Tracy and Robins, 2004). Guilt comes from transgressing a social or moral standard, while shame stems from a failure to live up to an ego ideal (e.g., Frijda et al., 1989). Accordingly, it is likely that negative gossip leads to higher self-conscious negative emotions than positive gossip. Furthermore, self-conscious negative emotions like guilt and shame induce tension, regret and a tendency to alter the situation (Solomon, 1993). This makes individuals less likely to repeat the negative behavior and more likely to repair the harm they have caused (Frijda et al., 1989). Indeed, research suggests that individuals who have violated norms or underperformed at work engage in compensatory behavior to mitigate their negative emotions and repair their social relations (Nelissen and Zeelenberg, 2009). Therefore, and in accordance with the social control function of gossip (Beersma and van Kleef, 2011; Beersma and Van Kleef, 2012; Feinberg et al., 2012), we propose the following:

Negative (relative to positive) gossip triggers repair intentions and this effect is mediated by self-conscious negative emotions (*hypothesis 1*).

Positive gossip entails a favorable evaluation (Foster, 2004). Because people need positive evaluations from others to function well (Sedikides and Strube, 1997), this is likely to please its targets. Because people are motivated to believe that the favorable evaluation is due to their own merit (Kunda, 1990), positive gossip is likely to result in positive self-conscious emotions (such as pride; see Lazarus, 1991; Solomon, 1993; Tracy and Robins, 2004; Tracy et al., 2010). Pride makes people feel that they deserve high status and group acceptance and also signals to others that they have high personal value (Tracy et al., 2010). However, expressions of pride are likely to manifest subtly in an interpersonal interaction. Lazarus (1991) notes that it is difficult to specify a clear action tendency for pride, because self-praise is socially undesirable. Therefore, we do not predict specific behavioral intentions for positive gossip targets who feel positive self-conscious emotion, but we predict that:

Positive gossip generates higher self-conscious positive emotion than negative gossip (*hypothesis 2)*.

### Other-Directed Emotional Reactions and Behavioral Intentions

Self-conscious emotions may, however, not be the only emotional reaction to gossip. Gossip communicates information and evaluations about targets to third parties, and shapes targets' reputation among group members in a positive or negative way (e.g., Burt, 2008; Beersma and Van Kleef, 2012; Feinberg et al., 2012). Individuals' reputation influences the extent to which they are socially accepted and trusted, or ostracized from groups (Feinberg et al., 2014), which is fundamental for well-being. Based on the AET, we propose that individuals are likely to react affectively to events that shape their reputation and to act to protect or promote their reputation. Because gossipers are the agents that influence targets' reputation, gossip is also likely to generate other-directed positive or negative emotions.

Negative gossip informs targets that the gossipers criticized them and exposed their weaknesses to others. Because it takes place in their absence, targets may experience negative gossip about themselves as offensive and unfair and may hold the gossiper responsible for harming them (Lazarus, 1991; Solomon, 1993). Gossipers may indeed abusively self-enhance at the expense of gossip targets (Wert and Salovey, 2004), by damaging their reputation (Burt, 2008), restraining their power (Ogasawara, 1998), or decreasing their sexual attractiveness (Massar et al., 2012). Targets of negative gossip are likely to feel that the gossiper has violated their interests and to experience other-directed negative emotion (e.g., anger, Lazarus, 1991). Because people strive for equitable relationships with co-workers (Adams, 1965), experiencing unfair treatment causes distress and desire for revenge (Lazarus, 1991). Anger facilitates punishing gossipers and justifies counter-attacks against offenders (Lazarus, 1991). Thus, we expect that:

Negative (relative to positive) gossip leads to retaliation intentions against the gossiper and this relationship is mediated by other-directed blame (*hypothesis 3*).

In contrast, positive gossip may generate positive emotions directed at the gossiper (i.e., interpersonal liking^1^), because targets will recognize the active role gossipers play in elevating their reputation (Sommerfeld et al., 2007; Burt, 2008). Positive gossip creates “cost free social rewards” (Merry, 1984) and people like others with whom they associate rewards and positive outcomes with (Sternberg, 1987). Moreover, behaviors that imply warmth and affiliation, such as positive gossip, tend to elicit reciprocal responses from others (Kiesler, 1983). Indeed, a meta-analysis showed that indirect ingratiating behaviors, such as other-enhancement, have strong effects on interpersonal attraction and liking (Gordon, 1996). As such, targets of positive gossip may experience higher other-directed happiness than targets of negative gossip. Furthermore, interpersonal liking helps individuals develop trust and establish collaborations due to a feeling of shared social identity (Hogg and Turner, 1985). Because people who are liked are attributed with favorable motives (Nicholson et al., 2001), positive gossip targets might think that they have shared values with the gossiper and will find a trustworthy ally in this person. Thus, we predict that:

Positive (relative to negative) gossip predicts targets' affiliation intentions with the gossiper due to increased other-directed positive emotions (*hypothesis 4)*.

### Gossip vs. Feedback

As gossip provides targets with evaluative information about themselves, it may serve as informal feedback. This raises the question whether targets' emotional and behavioral reactions to gossip are unique and can be distinguished from reactions to (formal) feedback, or whether those reactions to gossip are general response tendencies to positive and negative evaluations about the self.

Although gossip and formal feedback both contain self-relevant information for targets, they have a different audience and purpose and may therefore have different effects on targets' emotions and subsequent behavior. Feedback is addressed directly to its targets and is meant to help them improve (Kluger and DeNisi, 1996) and is usually embedded in organizational procedures and policies. However, gossip is spread in the targets' absence, with an obscure purpose [e.g., to gain information, protect others, harm targets, or for enjoyment, (Beersma and Van Kleef, 2012)], usually in spontaneous interactions between gossipers. Because feedback is addressed directly and usually obtained through formal channels, with the intention to help, feedback is likely to be perceived as more legitimate by targets than gossip. Therefore, we expect that:

Feedback leads to stronger self-conscious negative (*hypothesis 5a*) and positive emotions than gossip (*hypothesis 5b*).

Furthermore, because gossip is likely to be more threatening, more socially undesirable and more intrusive than feedback (Dunbar, 2004; Foster, 2004), we predict that:

Gossip leads to higher other-directed negative emotions (*hypothesis 6a*) and to lower other-directed positive emotions than feedback (*hypothesis 6b*).

### Predispositions

The AET contends that people's predispositions influence their attention to and processing of social and emotional stimuli and thus their emotional and behavioral responses to affective work events (Weiss and Cropanzano, 1996). Whereas, positive self-conscious (pride) and other-directed emotions (liking) can easily co-occur and may be universally experienced by targets of positive gossip, while individuals may react differently to negative gossip. Threats and losses trigger more specific responses than gains (Baumeister et al., 2001) and these responses may depend on dispositional factors (Mischel and Shoda, 1995). Specifically, we propose that responses to negative gossip will depend on individuals' core self-evaluations [CSE, (Judge et al., 1997)] and concern for reputation [CR, (De Cremer and Tyler, 2005)].

#### Core Self-Evaluations

People's sensitivity to negative stimuli may vary depending on their core self-evaluations [CSE, (Judge et al., 1997)]. CSE is a higher order construct that reflects individuals' fundamental appraisals about their self-worth and capabilities. Individuals with low CSE evaluate their identities and capabilities negatively and have low self-esteem, self-efficacy, internal locus of control, and emotional stability (Judge et al., 1997, 2003). Due to their weak coping and self-regulation skills, people with low CSE seek little social support, develop few social ties, are vulnerable to threats (Chang et al., 2012) and more intensely experience stressful work events, such as negative gossip (Grant and Sonnentag, 2010). Because targets of negative gossip with low CSE see themselves as unworthy individuals, unable to cope with challenges, they may think that indeed they are responsible for their negative evaluations. Thus, we hypothesize that:

The effect of gossip valence on self-conscious negative emotions is stronger for individuals with low rather than high CSE (*hypothesis 7a*).

The indirect effect of gossip valence on repair intentions through self-conscious negative emotions is stronger for individuals with low rather than high CSE (*hypothesis 7b*).

#### Reputational Concerns

Reputation is the extent to which someone is known to be trustworthy (Burt, 2008) and represents shared opinions about a focal person above and beyond directly observable behavior (Sommerfeld et al., 2007; Burt, 2008). People need a favorable reputation to ensure future collaborations with others (Burt, 2008), but their reputation is vulnerable to gossip (Foster, 2004). Concern for reputation (CR) reflects the importance people give to their public image of being trustworthy (De Cremer and Tyler, 2005). Gossip targets who are concerned about their reputation are highly aware of the losses associated with a bad reputation (Burt, 2008). Consequently, targets with high CR may perceive others who spread negative gossip about them as offensive and unfair, causing them harm. We therefore expect that:

The effect of gossip valence on other-directed negative emotions is stronger for people with high rather than low CR (*hypothesis 8a*).

The indirect effect of gossip valence on retaliation intentions through other-directed negative emotions is stronger for people with high rather than low CR (*hypothesis 8b*).

### Overview of Studies

**Figure 1:**
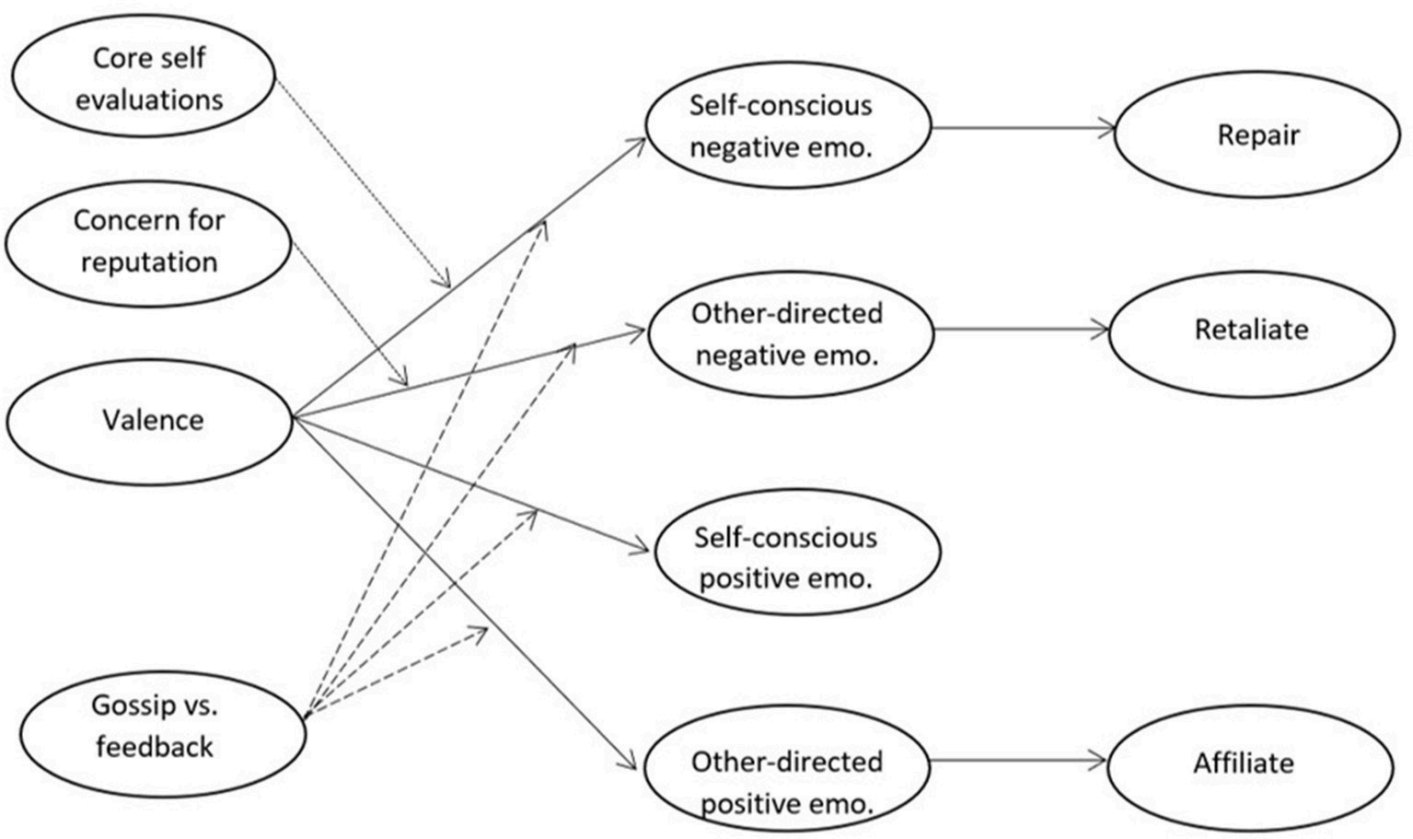
Theoretical model. Solid lines represent relationships tested in all three studies. Dashed lines represent relationships tested in Study 2. Dotted lines represent relationships tested in Study 3.

To test our hypotheses, we conducted three studies in which we manipulated the valence of gossip overheard by targets. We focused on performance-related gossip, which often occurs in the workplace or similar achievement environments. In Study 1, hypothesized mediation relationships between gossip valence, emotional reactions, and behavioral intentions (H1-4) were tested in a scenario experiment with a sample of university students. In Study 2, a scenario study among employees aimed to replicate these findings and additionally tested whether gossip and feedback differ in the emotional and behavioral reactions they elicit for gossip targets (H5-6). Study 3 was a critical incidents study that examined the hypothesized moderating effects of core self-evaluation and concern for reputation in targets' responses to positive and negative gossip (H7-8). Figure 1 presents the overview of our model.

## Study 1

### Method

#### Design and Participants

Two hundred and twenty six economics and business undergraduates at a Dutch university (108 female, *M*_age_ = 21.76, *SD* = 3.21), participated in exchange for a course credit or for 4 Euros. Participants were randomly assigned to a positive (*N* = 116) or negative (*N* = 110) gossip condition. The study was approved by a research ethics committee, and participants gave written informed consent.

#### Procedure

Upon arrival at the research laboratory, participants were seated in separate cubicles and informed that they would participate in research about students' reactions to informal evaluative talk about themselves. Participants read a scenario and were asked to imagine that they had written it.

#### Gossip Valence Manipulation

Participants imagined working on a group assignment and overhearing two classmates talk about them behind their back. Participants were targets of either *positive* or *negative* performance-related gossip: “A said to B that you *are/are not* a good group member and *likes/dislikes* working with you, because your contribution to the assignment is *remarkable/disappointing*. You seem to be very *hardworking/lazy*, since you *always/never* come prepared to the meetings and choose to do the *most difficult/easiest* tasks.” Next, participants filled in manipulation checks and dependent variable measures, were debriefed, compensated and thanked for participation^2^.

### Measures

#### Manipulation Checks

Participants were asked to indicate whether the overheard gossip was positive or negative. They could choose between (1) *The information classmate A told classmate B about me is positive* and (2) *The information classmate A told classmate B about me is negative*. Next, participants were asked to summarize the information they overheard.

#### Dependent Measures

For all dependent variables we used a 7-point Likert response with scales ranging from 1 (strongly disagree) to 7 (strongly agree).

#### Emotions

Emotions were measured using items from the PANAS-X scale (Watson and Clark, 1994). Specifically, participants indicated to what extent the overheard gossip information made them feel “guilty,” “ashamed,” “blameworthy,” “angry at self” (*self-conscious negative emotions*, α = 0.96), “proud,” “strong,” “bold” (*self-conscious positive emotions*, α = 0.91), “hostile,” “irritable,” and “angry” (*other-directed negative emotions*, α = 0.93). *Other-directed positive emotions* were measured with 3 items adapted from Wojciszke et al. (2009): “I like classmate A,” “I have warm feelings about classmate A,” and “I feel close to classmate A” (α = 0.93).

#### Behavioral Intentions

*Repair intentions* were measured with 3 items developed by Martinescu et al. (2014). Following the introductory phrase “The things classmate A said about me to classmate B would help me,” the specific items were: “Understand how to improve my contribution to the group assignment,” “Improve my performance in the group assignment,” and “Understand that I can do better in the group assignment” (α = 0.86). Behavioral intentions regarding the gossiper were measured following the introductory phrase: “How likely would it be for you to do the following things…” using two items for *retaliation intentions*: “Talk badly about classmate A” and “Punish classmate A if I can” (α = 0.74), and two items for *affiliation intentions*: “Team-up with classmate A in the future,” “Try to become friends with classmate A” (α = 0.76).

### Results

#### Manipulation Checks

One participant in the positive gossip condition and three in the negative condition incorrectly indicated the valence of overheard information. The summaries of overheard information were coded by the first author blind to the condition and a second coder, blind to the gossip condition and the hypotheses, coded a subset of 72 stories (37 in the positive condition). All stories (100%) were coded as matching the condition by both coders, thus the gossip valence manipulation was successful.

#### Descriptive Statistics

Table 1 presents means, standard deviations, and correlations for variables in Study 1. Gossip valence was strongly correlated with all other variables (see zero-order correlations in Table 1, below the diagonal), which may generate spurious correlations among emotions and behavioral intentions. Therefore, Table 1 also contains partial correlations among these variables, controlling for valence (above the diagonal). Partial correlations showed that self-conscious and other-directed negative emotions were unrelated (*r* = −0.05, ns), whereas self-conscious and other-directed positive emotions were positively related (*r* = 0.25, *p* < 0.01).

**Table 1:** Means, standard deviations, and zero-order Pearson correlations for variables in Study 1.

**Variable**	**Mean**	***SD***	**1**.	**2**.	**3**.	**4**.	**5**.	**6**.	**7**.	**8**.	**9**.	**10**.
1. Age	21.76	3.21	–	−0.01	–	−0.21^**^	0.02	0.13^*^	−0.09	−0.04	−0.02	0.02
2. Gender	−0.04	1.00	−0.01	–	–	0.06	0.03	−0.06	0.00	0.09	−0.17^**^	−0.01
3. Gossip valence	0.03	1.00	−0.10	0.01	–	–	–	–	–	–	–	–
4. SCNE	2.82	1.90	−0.05	0.03	−0.77^**^	–	−0.05	−0.32^**^	0.03	0.32^**^	−0.08	0.14^*^
5. ODNE	2.93	1.88	0.09	0.01	−0.80^**^	0.61^**^	–	−0.00	−0.29^**^	−0.10	0.47^**^	−0.18^**^
6. SCPE	3.79	1.99	−0.01	−0.02	0.85^**^	−0.77^**^	−0.69^**^	–	0.25^**^	−0.17^*^	0.17^**^	0.05
7. ODPE	3.32	1.68	−0.13^*^	0.01	0.72^**^	−0.54^**^	−0.70^**^	0.70^**^	–	0.16^*^	−0.18^**^	0.52^**^
8. Repair	4.14	1.72	0.00	0.07	−0.44^**^	0.52^**^	0.29^**^	−0.45^**^	−0.21^**^	–	−0.09	0.18^**^
9. Retaliate	2.24	1.43	0.04	−0.14^*^	−0.61^**^	0.43^**^	0.71^**^	−0.45^**^	−0.54^**^	0.20^**^	–	−0.06
10. Affiliate	3.39	1.62	−0.04	−0.00	0.57^**^	−0.36^**^	−0.55^**^	0.50^**^	0.71^**^	−0.11	−0.39^**^	–

N = 226;

* p < 0.05;

** *p < 0.01; gender was coded −1 for males and 1 for females; gossip valence was coded 1 for positive condition and −1 for negative condition; SCNE, self-conscious negative emotions; ODNE, other-directed negative emotions; SCPE, self-conscious positive emotions; ODPE, other-directed positive emotions; zero order correlations below the diagonal and partial correlations controlling for gossip valence above the diagonal*.

#### Hypotheses Testing

To test our hypotheses, we conducted multiple regressions with the Preacher et al.'s (2007) procedure for mediation analysis. To assess the indirect effect of gossip valence on each of the three behavioral intentions we employed 5,000 bootstrap samples; all four types of emotional responses were included as simultaneous mediators and we examined the unique indirect effects for each emotion type (see Table 2).

**Table 2:** Indirect effect analyses in Study 1.

	**Mediator variable models**			
	**SCNE**	**ODNE**	**SCPE**	**ODPE**
	***b (t)***	***b (t)***	***b (t)***	***b (t)***
Gossip valence	−1.48^***^ **(−18.58)**	−1.52^***^ **(−20.57)**	1.69^***^ **(24.32)**	1.21^***^ **(15.72)**
		**Dependent variable models**		
		**Repair intentions**	**Retaliation intentions**	**Affiliation intentions**
		***b (t)***	***b (t)***	***b (t)***
Gossip valence		−0.28 (−1.18)	−0.44^*^ (−2.74)	0.43^*^ (2.32)
SCNE (self-conscious negative emotions)		0.36^***^ **(4.20)**	0.0037 (0.06)	0.12 (1.82)
ODNE (other-directed negative emotions)		−0.06 (−0.66)	0.44^***^ **(7.23)**	−0.02 (−0.30)
SCPE (self-conscious positive emotions)		−0.18 (−1.80)	0.21^**^ (3.16)	−0.06 (−0.78)
ODPE (other-directed positive emotions)		0.23^*^ (2.56)	−0.09 (−1.60)	0.60^***^ **(8.53)**
		**Indirect effects**		
SCNE		**−** **0.53 [−** **0.82;** **−** **0.28]**	−0.00 [−0.23; 0.23]	−0.18 [−0.38; 0.009]
ODNE		−0.09 [−0.20; 0.36]	**−0.68 [−0.95;** **−0.43]**	0.03 [−0.18; 0.29]
SCPE		−0.31 [−0.67; 0.03]	0.37 [.09; 0.62]	−0.10 [−0.37; 0.15]
ODPE		0.28 [.07; 0.50]	−0.12 [−0.28; 0.01]	**0.73 [.53; 0.94]**

N = 226;

* p < 0.05;

** p < 0.01;

*** *p < 0.001; coefficients in boldface represent hypothesized effects*.

Targets of negative gossip experienced higher self-conscious negative emotions (*M* = 4.35; *SD* = 1.54) than targets of positive gossip (*M* = 1.35; *SD* = 0.73), *b* = −1.48, *p* < 0.001. Self-conscious negative emotions were positively related to repair intentions, *b* = 0.36, *p* < 0.001, and mediated the effect of gossip valence on repair intentions, *indirect effect* = −0.53, 95% CI [−0.82; −0.28], supporting hypothesis 1. As predicted by hypothesis 2, positive gossip targets experienced higher self-conscious positive emotions (*M* = 5.44; *SD* = 0.92) than negative gossip targets (*M* = 2.05; *SD* = 1.15), *b* = 1.69, *p* < 0.001.

Furthermore, negative gossip targets experienced higher other-directed negative emotions (*M* = 4.49; *SD* = 1.41) than positive gossip targets (*M* = 1.44; *SD* = 0.71), *b* = −1.52, *p* < 0.001. Other-directed negative emotions positively predicted retaliation intentions, *b* = 0.44, *p* < 0.001, and mediated the effect of gossip valence on retaliation intentions, *indirect effect* = −0.68, 95% CI [−0.95; −0.43], supporting hypothesis 3. Finally, positive gossip targets experienced higher positive other-directed emotions (*M* = 4.51; *SD* = 1.28) than negative gossip targets (*M* = 2.07; *SD* = 1.02), *b* = 1.21, *p* < 0.001. Other-directed positive emotions predicted affiliation intentions, *b* = 0.60, *p* < 0.001, and mediated the effect of gossip valence on affiliation intentions, *indirect effect* = 0.73, 95% CI [0.53;0.94], supporting hypothesis 4.

In addition to the hypothesized indirect effects, the analyses reported in Table 2 showed that other-directed positive emotion also predicted repair intentions and mediated the effect of gossip valence on repair intentions. Furthermore, self-conscious positive emotion positively predicted retaliation intentions, and mediated the effect of gossip valence on retaliation intentions. Although these indirect effects were unexpected, they were weaker than the hypothesized effects.

### Discussion

Results of Study 1 supported our expectations that gossip targets experience distinct emotions which predict specific behavioral intentions. Specifically, self-conscious negative emotions were higher for targets of negative than for targets of positive gossip, and generated repair intentions. Negative compared to positive gossip also evoked stronger other-directed negative emotions, which increased retaliation intentions against gossipers. Compared to negative gossip, positive gossip made targets feel stronger positive self-conscious emotions, as well as stronger other-directed positive emotions, which increased affiliation intentions. In Study 1 we thus found good support for our predictions that gossip about the self, evokes positive and negative emotions and behavioral intentions. In a second scenario study we investigated whether people have different reactions to gossip and feedback.

## Study 2

### Method

#### Participants and Design

Two hundred and four U.S. employees who worked at least 20 h a week, completed an online questionnaire via MTurk in exchange for 1.5 $. Three participants failed an attention check that asked them to not indicate their hobbies and were excluded from the analyses. The remaining 201 participants, 73 females, had an average age of 34.11 years (*SD*_age_ = 9.43). The study had a 2 (gossip valence: positive vs. negative) x 2 (type of information: gossip *vs*. feedback) between-subjects design. The study was approved by a research ethics committee, and participants gave written informed consent.

#### Procedure

Participants read that the study was about workplace communication and received a scenario in which they work at a sales department with two others, Sam and Alex, who have similar jobs.

#### Manipulations

Participants imagined that while reading emails in their office, they either receive formal feedback about their work contribution, as part of an anonymous 360-degree feedback system, which they recognize as written by Sam, or that they accidentally overhear Sam gossip to Alex about them. The information participants received was the same in the feedback and gossip conditions and was framed either negatively or positively: “[Target name] knows we have to focus on the new clients *and/but* I have the feeling that (s)he *is/is not* working very hard lately. That's *really/not* nice, because we all need to take more responsibility.”

### Measures

#### Manipulation Checks

Participants indicated whether the received information was positive or negative. They could choose between: *My colleague Sam* (1) *said something negative about me* and (2) *said something positive about me*. Next, participants indicated whether the information about them was in the form of feedback or gossip: *The things Sam said about me (1) were meant to be communicated as feedback to me* and (2) *were meant to be communicated behind my back*.

#### Emotions and Behavioral Intentions

Rather than using the PANAS-X scale to measure emotions, we developed items that better distinguish self-conscious vs. other-directed emotions. Because *self-conscious negative emotions* were assessed with clear self-referenced items in Study 1 (e.g., “I feel guilty”), we did not alter that scale (α = 0.98). We measured *self-conscious positive emotions* with three items: “Happy about myself,” “Enthusiastic about myself,” and “Proud,” α = 0.98, *other-directed negative emotions* with two items: “I feel angry with my colleagues” and “I feel upset with my colleagues” (α = 0.99), and *other-directed positive emotions* were measured using the items: “I feel happy about my colleagues,” “I feel enthusiastic about my colleagues,” “I like my colleagues,” and “I feel close to my colleagues,” α = 0.97.

In Study 1, *repair intentions* were measured indirectly, by asking how helpful the gossip would be for improving the targets' performance. In Study 2, we used a more direct measure (2 items), reflecting increased work effort: “…Try to do my job better” and “…Put in more effort at work,” α = 0.96. *Retaliation intentions*, α = 0.85, and *affiliation intentions*, α = 0.94, were measured as in Study 1.

### Results

#### Manipulation Checks

One participant in the positive and one in the negative gossip condition incorrectly indicated valence of the information. Furthermore, 13 participants who received feedback and 11 who received gossip incorrectly indicated the type of information. In total, 23 participants failed manipulation checks and were excluded from further analyses.

#### Descriptive Statistics

Gossip valence was strongly correlated with the other variables (see zero-order correlations in Table 3, below the diagonal), which may lead to spurious correlations among emotions and intentions. Therefore, Table 3 also contains partial correlations among these variables, controlling for valence (above the diagonal). Partial correlations showed that negative self-conscious and other-directed emotions were unrelated (*r* = 0.03, ns), whereas self-conscious and other-directed positive emotions were positively related (*r* = 0.66, *p* < 0.01). This correlation pattern is consistent with results of Study 1.

**Table 3:** Means, standard deviations, and correlations for variables in Study 2.

	**Mean**	***SD***	**1**.	**2**	**3**.	**4**.	**5**.	**6**.	**7**.	**8**.	**9**.	**10**.	**11**.
1. Age	34.15	9.27	–	−0.08	–	−0.02	−0.05	0.16^*^	−0.00	−0.04	−0.06	−0.05	−0.06
2. Gender	−0.28	0.96	−0.08	–	–	−0.11	0.03	0.17^*^	0.15^*^	0.04	0.10	−0.11	0.04
3. Info. valence	0.02	1.00	0.06	−0.01	–	–	–	–	–	–	–	–	–
4. Info. type	−0.03	1.00	−0.02	−0.11	0.04	–	0.21^**^	−0.15^*^	0.03	0.18^*^	0.01	−0.09	0.02
5. SCNE	2.43	1.92	−0.08	0.04	−0.69^**^	0.12	–	0.03	−0.17^*^	0.06	0.39^**^	0.00	0.22^**^
6. ODNE	2.78	2.08	0.05	0.11	−0.79^**^	−0.13	0.56^**^	–	0.01	−0.27^**^	−0.12	0.32^**^	−0.18^*^
7. SCPE	3.84	2.16	0.05	0.07	0.84^**^	0.05	−0.65^**^	−0.66^**^	–	0.66^**^	0.22^**^	0.07	0.35^**^
8. ODPE	3.87	2.00	0.02	0.00	0.84^**^	0.14	−0.55^**^	−0.75^**^	0.90^**^	–	0.39^**^	−0.04	0.62^**^
9. Repair	5.43	1.27	−0.05	0.10	0.07	0.01	0.23^**^	−0.13	0.18^*^	0.27^**^	–	−0.24^**^	0.42^**^
10. Retaliate	1.79	1.33	−0.07	−0.09	−0.38^**^	−0.10	0.27^**^	0.48^**^	−0.28^**^	−0.34^**^	−0.25^**^	–	−0.02
11. Affiliate	4.25	1.85	−0.01	0.02	0.64^**^	0.05	−0.32^**^	−0.59^**^	0.69^**^	0.80^**^	0.37^**^	−0.26^**^	–

N = 178;

* p < 0.05;

** *p < 0.01; gender was coded −1 for males and 1 for females; information valence was coded 1 for positive condition and −1 for negative condition; type of information was coded with −1 for gossip and 1 for feedback condition; SCNE, self-conscious negative emotions; ODNE, other-directed negative emotions; SCPE, self-conscious positive emotions; ODPE, other-directed positive emotions; zero order correlations below the diagonal and partial correlations controlling for gossip valence above the diagonal*.

#### Main Findings

**Figure 2:**
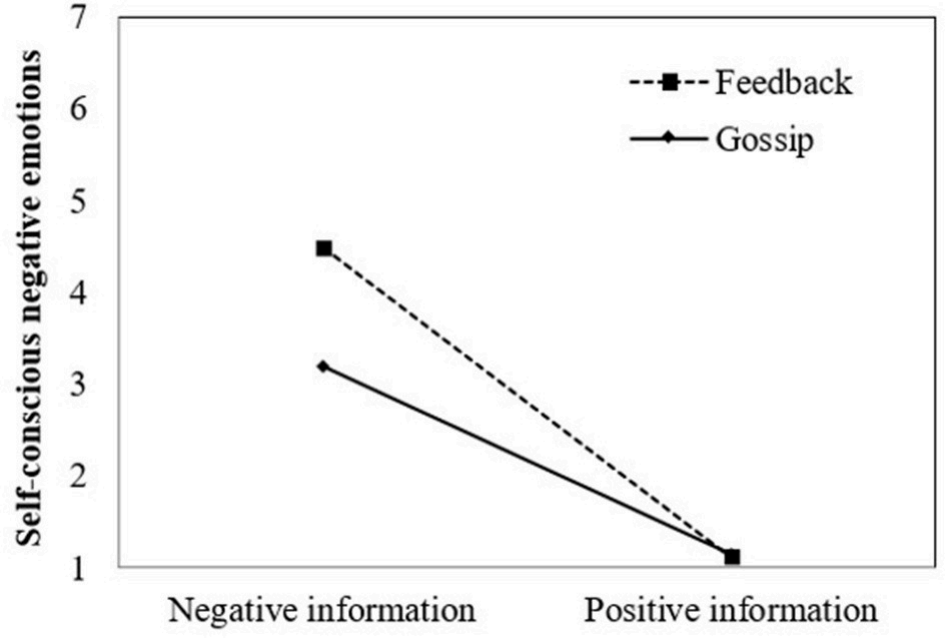
Self-conscious negative emotions as a function of valence and type of information in Study 2.

We tested whether the effects of gossip, found in Study 1, were distinct from the effects of feedback, with a bootstrapping procedure for assessing indirect and conditional indirect effects (Preacher et al., 2007). Results showed that participants who received negative information about themselves experienced higher self-conscious negative emotions (*M* = 3.79, *SD* = 1.94) than participants who received positive information (*M* = 1.13, *SD* = 0.45), *b* = −1.35, *p* < 0.001, and that participants who received feedback (*M* = 2.68, *SD* = 1.77) experienced higher self-conscious negative emotions than participants who received gossip (*M* = 2.20, *SD* = 2.06), *b* = 0.31, *p* < 0.01, consistent with hypothesis 5a. These main effects were qualified by an interaction effect showing that feedback targets had higher self-conscious negative emotions than gossip targets when the information was negative, but not when it was positive, *b* = −0.33, *p* < 0.01, as shown in Figure 2. Furthermore, self-conscious negative emotions predicted repair intentions, *b* = −0.36, *p* < 0.001. Consequently, the indirect effect of information valence on repair intentions through self-conscious negative emotions was stronger when participants received feedback (*indirect effect* = −0.60 [−0.88; −0.35]) rather than gossip (*indirect effect* = −0.36 [−0.57; −0.20]). These findings support hypothesis 1, and in addition show that people experienced higher self-conscious negative emotions in response to negative feedback than to negative gossip, as shown in Table 4.

**Table 4:** Indirect effects and conditional indirect effects analyses in Study 2.

	**Mediator models**			
	**SCNE**	**ODNE**	**SCPE**	**ODPE**
	**b *(t)***	**b *(t)***	**b *(t)***	**b *(t)***
Information valence (IV)	−1.35 (−13.60)^***^	−1.63 (−17.34)^***^	1.82 (20.89)^***^	1.67 (20.64)^***^
Type of information (TI)	0.31 (3.11)^**^	−0.19 (−2.11)^*^	0.03 (0.45)	0.20 (2.52)^*^
IV^*^TI	−0.33 (−3.32)^**^	0.09 (1.04)	0.03 (0.41)	−0.02 (−0.35)
		**Dependent variable models**		
		**Repair intentions**	**Retaliation intentions**	**Affiliation intentions**
		***b (t)***	***b (t)***	***b (t)***
Information valence (IV)		−0.30 (−1.56)	−0.12 (−0.56)	0.11 (0.55)
SCNE (self-conscious negative emotions)		0.36 (5.88)^***^	0.01 (0.16)	0.18 (2.93)^**^
ODNE (other-directed negative emotions)		−0.06 (−0.86)	0.31 (4.08)^***^	−0.01 (−0.17)
SCPE (self-conscious positive emotions)		0.11 (1.15)	0.08 (0.77)	−0.04 (−0.41)
ODPE (other-directed positive emotions)		0.33 (2.99) ^**^	−0.009 (−0.58)	0.82 (7.23)^***^
		**Indirect effects**		
SCNE		**−0.47 [−0.72;** **−0.30]**	−0.01 [−0.25; 0.26]	−0.24 [−0.47; −0.04]
ODNE		0.09 [−0.13; 0.35]	**−0.51 [−0.86;** **−0.16]**	0.02 [−0.30; 0.35]
SCPE		0.21 [−0.12; 0.59]	0.15 [−0.20; 0.58]	−0.07 [−0.49; 0.29]
ODPE		0.56 [.17; 0.87]	−0.01 [−0.41; 0.32]	**1.38 [0.96; 1.89]**
**Mediator**	**Moderator**	**Conditional indirect effect**		
SCNE	TI Gossip	**−0.36 [−0.57;** **−0.20]**		
	Feedback	**−0.60 [−0.88;** **−0.35]**		

N = 178;

* p < 0.05;

** p < 0.01;

*** *p < 0.001; information valence was coded 1 for positive condition and −1 for negative condition; type of information was coded with −1 for gossip and 1 for feedback condition; coefficients in boldface represent hypothesized effects*.

Receiving positive information about oneself elicited higher self-conscious positive emotions (*M* = 5.63, *SD* = 1.19), than receiving negative information (*M* = 1.97, *SD* = 1.11), *b* = 1.82, *p* < 0.001, but there was no effect of type of information, *b* = 0.03, ns, and no interaction effect on self-conscious positive emotions, *b* = 0.03, ns. These results support hypothesis 2, but not hypothesis 5b: positive information makes targets feel better about themselves than negative information, but there is no difference between gossip and feedback.

Receiving negative information about the self, predicted higher other-directed negative emotions (*M* = 4.47, *SD* = 1.72) than positive information (*M* = 1.17, *SD* = 0.55), *b* = −1.63, *p* < 0.001, and receiving gossip (*M* = 3.04, *SD* = 2.21) predicted higher other-directed negative emotions than receiving feedback (*M* = 2.50, *SD* = 1.89), *b* = −0.19, *p* < 0.05. The interaction effect of valence and information type on other-directed negative emotions was not significant. Furthermore, other-directed negative emotions predicted retaliation intentions, *b* = 0.31, *p* < 0.001, and mediated the effect of information valence on retaliation intentions, *indirect effect* = −0.51 [−0.86; −0.16]. These results support hypotheses 3 and 6a and show that people reacted with higher other-directed negative emotions to gossip than to feedback.

Participants experienced higher other-directed positive emotions in response to positive information (*M* = 5.52, *SD* = 1.09), then in response to negative information (*M* = 2.15, *SD* = 1.08), *b* = 1.67, *p* < 0.001, and feedback generated higher other-directed positive emotions (*M* = 4.16, *SD* = 1.95) than gossip (*M* = 3.60, *SD* = 2.02), *b* = 0.20, *p* < 0.05. There was no interaction effect of information type and valence on other-directed positive emotions, *b* = −0.02, ns. Furthermore, other-directed positive emotions predicted affiliation intentions, *b* = 0.82, *p* < 0.001, and mediated the effect of information valence on affiliation intentions, *indirect effect* = 1.38 [0.96; 1.89]. These results support hypotheses 4 and 6b.

In addition to the hypothesized indirect effects, the analyses reported in Table 4 showed that other-directed happiness predicted repair intentions and mediated the effect of information valence on repair intentions. Moreover, self-conscious negative emotions predicted affiliation intentions, and mediated the effect of information valence on affiliation intentions.

### Discussion

The findings of Study 2 are consistent with results of Study 1, and in addition indicate that people have distinguishable reactions to feedback and gossip about the self. People who received feedback experienced stronger self-conscious negative emotions than people who received gossip, but only when the information was negative, possibly because they attributed higher responsibility to themselves. Negative information generated higher self-conscious negative emotions and repair intentions than positive information, especially when framed as feedback rather than gossip. The two types of self-relevant information generated similar self-conscious positive emotion, but people experienced stronger other-directed negative emotions and weaker other-directed positive emotions in the gossip condition than in the feedback condition. As in Study 1, negative compared to positive information evoked more other-directed negative emotions, which increased retaliation intentions. Compared to negative gossip, positive gossip induced higher self-conscious and other-directed positive emotions in gossip targets, which increased affiliation intentions. Study 3 further extend these findings and examines the moderating effects of CSE and CR on negative emotions in response to gossip using a critical incident study among Dutch employees.

## Study 3

### Method

#### Participants and Design

Two hundred and forty Dutch employees (*M*_age_ = 37.04, *SD*_age_ = 13.23; 109 female, 5 unspecified) working in different sectors (e.g., media, retail, tourism, health, banking, resource exploitation, public administration) voluntarily and anonymously completed an online survey, distributed using a snowballing method, with a response rate of 61.38%. Participants' current employment duration was on average 8.39 years (*SD*_employment_ = 9.44); 102 participants reported working part-time, with an average of 24.09 (*SD* = 8.12) hours per week. Most participants (73.2%) had a university degree, 21.7% had a vocational degree and 5.1% had a lower education degree. Participants were randomly assigned to a positive (*N* = 121) or negative (*N* = 119) gossip condition. Participants gave written informed consent.

#### Procedure

Participants were informed that the study was about workplace informal talk and interpersonal perception. First participants completed personality questionnaires. Next, they were randomly assigned to a gossip valence condition, and were asked to recall and describe a specific situation in which a co-worker said something either positive or negative about their work performance behind their back to another co-worker. Afterwards, they completed dependent measures, were debriefed and thanked for participation. The survey was translated into Dutch and back-translated into English. Participants could choose between a Dutch and an English version of the study.

#### Measures

CSE was measured using the 12-item *core self-evaluation* scale (Judge et al., 2003) and the validated Dutch translation of that scale (De Pater et al., 2007). This scale consists of the sub-dimensions of self-esteem (e.g., “Overall, I am satisfied with myself”), generalized self-efficacy (e.g., “When I try, I generally succeed”), neuroticism (e.g., “Sometimes when I fail I feel worthless”), and internal locus of control (e.g., “I determine what will happen in my life”); overall α = 0.85. *Concern for reputation* (CR; α = 0.73) was measured with seven items [e.g., “I wish to have a good reputation”, (De Cremer and Tyler, 2005)].

We used the same measures as in Study 2 to assess self-conscious negative emotions (α = 0.93), self-conscious positive emotions (α = 0.96), other-directed negative emotions (α = 0.91), and other-directed positive emotions (α = 0.88), and the behavioral intentions to repair (α = 0.92), retaliate, (α = 0.85), and affiliate (α = 0.88).

### Results

#### Manipulation Checks

Sixty-six participants (27.5%) were unable to recall being the target of either positive (*N* = 26) or negative gossip (*N* = 40), according to their condition, and were excluded from further analyses. Two coders blind to conditions assessed the valence of gossip incidents reported by participants, with an agreement of 0.87 (Cohen's Kappa). Two participants described gossip situations that were inconsistent with their assigned gossip valence condition and were excluded from the analysis; the final sample included 95 participants in the positive gossip condition and 77 in the negative gossip condition.

#### Descriptive Statistics

Table 5 presents means, standard deviations, and correlations for variables in Study 3. As in Study 1 and 2, most measures are strongly correlated with gossip valence, and therefore Table 5 also reports partial correlations controlling for gossip valence. These partial correlations showed that the negative self-conscious and other-directed emotions were correlated, *r* = 0.29, *p* < 0.01, and so were the positive self-conscious and other-directed emotions, *r* = 0.49, *p* < 0.01.

**Table 5:** Means, standard deviations, and zero-order Pearson correlations for variables in Study 3.

**Variable**	**Mean**	***SD***	**1**.	**2**.	**3**.	**4**.	**5**.	**6**.	**7**.	**8**.	**9**.	**10**.	**11**.	**12**.
1. Age	36.34	12.98	–	−0.21^*^	0.18^*^	−0.14		−0.13	−0.05	−0.07	0.03	−0.15	−0.22^*^	−0.21^*^
2. Gender	1.55	0.49	−0.22^*^	–	−0.12	0.11	–	−0.00	0.10	0.02	0.01	0.07	0.06	0.05
3. Core self-evaluations	5.28	0.79	0.18^*^	−0.12	–	−0.29^**^	–	−0.39^**^	−0.19^*^	0.15^*^	0.15^*^	−0.28^**^	−0.28^**^	−0.22^**^
4. Concern for reputation	4.49	0.91	−0.14	0.11	−0.31^**^	–	–	0.12	0.24^**^	0.11	0.07	0.28^**^	0.07	0.25^**^
5. Gossip valence	0.10	0.99	−0.07	0.16^*^	0.00	0.01	–	–	–	–	–	–	–	–
6. SCNE	1.69	1.11	−0.09	−0.07	−0.37^**^	0.11	−0.39^**^	–	0.29^**^	−0.18^*^	−0.09	0.30^**^	0.22^**^	0.18^*^
7. ODNE	2.10	1.56	0.00	−0.00	−0.17^*^	0.19^**^	−0.55^**^	0.44^**^	–	−0.003	−0.27^**^	−0.01	0.44^**^	0.17
8. SCPE	4.35	1.73	−0.10	0.11	0.13	0.08	0.60^**^	−0.37^**^	−0.33^**^	–	0.49^**^	0.05	0.08	0.16^*^
9. ODPE	4.38	1.55	−0.02	0.10	0.13	0.05	0.59^**^	−0.30^**^	−0.51^**^	0.67^**^	–	0.18^*^	−0.21^**^	0.24^**^
10. Repair	3.55	1.89	−0.13	0.03	−0.27^**^	0.27^**^	−0.23^**^	0.36^**^	0.12	−0.10	0.00	–	0.06	0.41^**^
11. Retaliate	1.75	1.18	−0.17	−0.01	−0.26^**^	0.06	−0.43^**^	0.36^**^	0.57^**^	−0.20^**^	−0.41^**^	0.16^*^	–	0.04
12. Affiliate	2.70	1.60	−0.22^*^	0.05	−0.22^**^	0.25^**^	0.00	0.16^*^	0.14	0.13	0.19^*^	0.40^**^	0.03	–

N = 172;

* p < 0.05;

** *p < 0.01; gender was coded −1 for males and 1 for females; gossip valence was coded 1 for positive condition and −1 for negative condition; SCNE, self-conscious negative emotions; ODNE, other-directed negative emotions; SCPE, self-conscious positive emotions; ODPE, other-directed positive emotions; zero order correlations below the diagonal and partial correlations controlling for gossip valence above the diagonal*.

#### Hypotheses Testing

We tested our hypotheses using a bootstrapping procedure for assessing indirect and conditional indirect effects (Preacher et al., 2007). Entering gossip valence as independent variable and all four emotions as mediator variables, we tested two moderated mediation models: the first had CSE as first-stage moderator and repair intention as outcome variable, while the second had CR as first stage moderator and retaliation intention as outcome. In addition, we tested a mediation model with affiliation intention as outcome variable. For each of the following models 5,000 bootstrap samples were used. Results are shown in Table 6.

**Table 6:** Indirect effects and conditional indirect effects analyses in Study 3.

	**Mediator models**			
	**SCNE**	**ODNE**	**SCPE**	**ODPE**
	***b (t)***	***b (t)***	***b (t)***	***b (t)***
Gossip valence (GV)	−0.44^***^ **(−5.55)**	−0.87^***^ **(−8.54)**	1.05^***^ **(9.77)**	0.91^***^ **(9.39)**
Gossip valence (GV)	−1.70^***^ **(−3.33)**	**0.30 (0.60)**		
Core self-evaluation (CSE)	−0.46^***^ (−4.88)			
GV^*^CSE	0.23^*^ **(2.50)**			
Concern for reputation (CR)		0.34^**^ (3.16)		
GV^*^CR		−0.26^*^ **(−2.40)**		
		**Dependent variable models**		
		**Repair intentions**	**Retaliation intentions**	**Affiliation intentions**
		***b (t)***	***b (t)***	***b (t)***
Gossip valence		−0.53^**^ (−2.73)	−0.21^*^ (−2.04)	−0.05 (−0.30)
SCNE (self-conscious negative emotions)		0.61^***^ **(4.43)**	0.15^*^ (2.03)	0.24^*^ (2.00)
ODNE (other-directed negative emotions)		−0.08 (−0.78)	0.27^***^ **(4.41)**	0.26^**^ (2.69)
SCPE (self-conscious positive emotions)		0.03 (0.29)	0.16^**^ (2.66)	0.05 (0.52)
ODPE (other-directed positive emotions)		0.26^*^ (2.04)	−0.18^**^ (−2.62)	0.37^**^ **(3.26)**
		**Indirect effects**		
SCNE		**−0.27 [−0.44;** **−0.15]**	−0.06 [−0.15; 0.01]	−0.10 [−0.21; −0.02]
ODNE		0.07 [−0.09; 0.28]	**−0.23 [−0.41;** **−0.09]**	−0.22 [−0.41; −0.05]
SCPE		0.03 [−0.22; 0.33]	0.17 [.01; 0.34]	0.05 [−0.13; 0.26]
ODPE		0.24 [−0.007; 0.50]	−0.17 [−0.35; −0.03]	**0.34 [0.16; 0.55]**
**Mediator**	**Moderator**		**Conditional indirect effects**	
SCNE CSE	Low (*M* −1 *SD*)	**−0.38 [−0.67;** **−0.18]**		
	High (*M* +1 *SD*)	**−0.15[−0.27;** **−0.06]**		
ODNE CR	Low (*M* −1 *SD*)		**−0.17 [−0.33;** **−0.07]**	
	High (*M* +1 *SD*)		**−0.30 [−0.53;** **−0.11]**	

N = 172;

* p < 0.05;

** p < 0.01;

*** *p < 0.001; gossip valence was coded 1 for positive condition and −1 for negative condition; coefficients in boldface represent hypothesized effects*.

**Figure 3:**
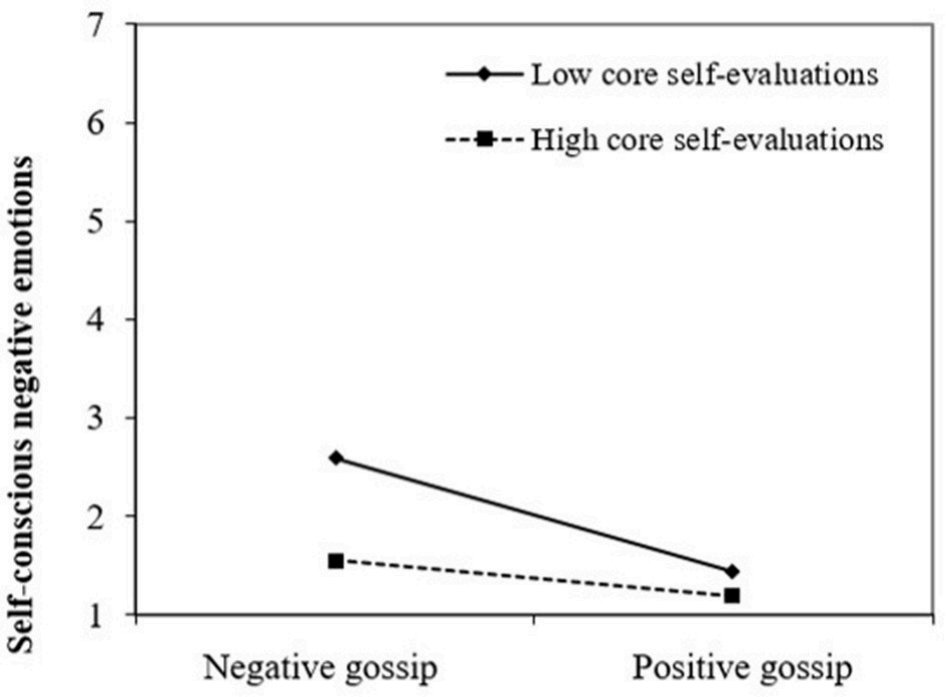
Self-conscious negative emotions as a function of gossip valence and core self-evaluations.

As predicted by hypothesis 1, self-conscious negative emotions were higher in the negative (*M* = 2.18, *SD* = 1.38) than in the positive gossip condition (*M* = 1.29, *SD* = 0.59), *b* = −1.70, *p* < 0.001. Consistent with hypothesis 7a, this effect was qualified by a significant interaction with CSE, *b* = 0.23, *p* < 0.05, showing that the effect of gossip valence on self-conscious negative emotions was stronger for participants with low CSE (*M* – 1 *SD*; *b* = −0.62, *t* = −6.09, *p* < 0.001) than for participants with high CSE (*M* + 1 *SD*; *b* = −0.25, *t* = −2.48, *p* < 0.05), as shown in Figure 3. Furthermore, self-conscious negative emotions were related to repair intentions, *b* = 0.61, *p* < 0.001. Consequently, and consistent with hypothesis 7b, the indirect effect of gossip valence on repair intentions through self-conscious negative emotions was stronger for participants with low CSE (*indirect effect* = −0.38, 95% CI [−0.67; −0.18]), than for participants with high CSE (*indirect effect* = −0.15, 95% CI [−0.27; −0.06]).

Furthermore, in line with hypothesis 2, positive gossip targets experienced higher self-conscious positive emotions (*M* = 5.31; *SD* = 1.12) than negative gossip targets (*M* = 3.21; *SD* = 1.64), *b* = 1.05, *p* < 0.001.

**Figure 4:**
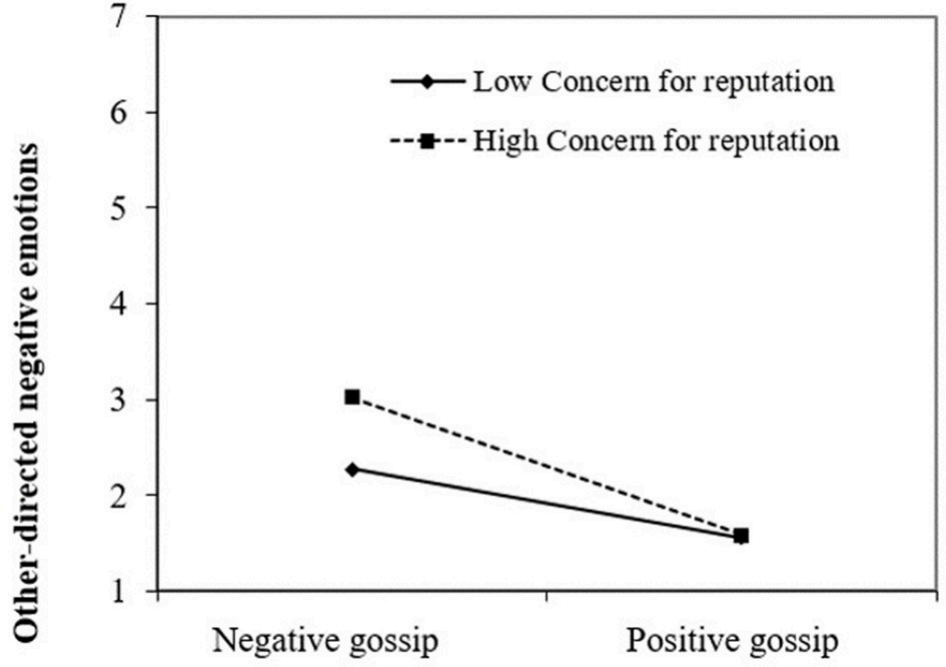
Other-directed negative emotions as a function of gossip valence and concern for reputation.

Supporting hypothesis 3, other-directed negative emotions were higher in the negative (*M* = 3.05, *SD* = 1.79) than in the positive gossip condition (*M* = 1.31, *SD* = 0.65), *b* = −0.87, *p* < 0.001. When CR was entered as a moderator, the effect of gossip valence on other-directed negative emotions became non-significant, *b* = 0.30, ns. However, consistent with hypothesis 8a, the interaction between gossip valence and CR was significant and in the hypothesized direction, *b* = −0.26, *p* < 0.05. Specifically, the effect of gossip valence on other-directed negative emotions was stronger for participants with high CR (*M* + 1 *SD*; *b* = −1.14, *t* = 8.15, *p* < 0.001) than for participants with low CR (*M* – 1 *SD*; *b* = −0.63, *t* = −4.63, *p* < 0.001), as illustrated in Figure 4. Furthermore, other-directed negative emotions predicted retaliation intentions, *b* = 0.27, *p* < 0.001. Consequently, and consistent with hypothesis 8b, the indirect effect of gossip valence on retaliation intentions through other-directed negative emotions was stronger for participants with high CR (*indirect effect* = −0.30, 95% CI [−0.53; −0.11]), than for participants with low CR (*indirect effect* = −0.17, 95% CI [−0.33; −0.07]).

Finally, as predicted by hypothesis 4, positive gossip targets experienced higher other-directed positive emotions (*M* = 5.22; *SD* = 1.21) than negative gossip targets (*M* = 3.38; *SD* = 1.30), *b* = 0.91, *p* < 0.001. Other-directed positive emotions predicted affiliation intentions, *b* = 0.37, *p* < 0.01, and mediated the effect of gossip valence on affiliation intentions, *indirect effect* = 0.34, 95% CI [0.16;0.55].

Additionally, the analyses reported in Table 6 showed that self-conscious positive emotions also predicted retaliation intentions and mediated the effect of gossip valence on retaliation intentions; other-directed positive emotions were negatively related to retaliation intentions and mediated the effect of gossip valence on retaliation intentions. Moreover, self-conscious negative emotions were related to affiliation intentions, and mediated the effect of gossip valence on affiliation intentions; other-directed negative emotions were related to affiliation intentions and mediated the effect of gossip valence on affiliation intentions. As in Study 1 and 2, these unpredicted indirect effects were weaker than hypothesized effects.

## General Discussion

Previous research has documented the role of gossip in maintaining cooperation in groups but has not investigated how people feel and behave when they actually encounter gossip about themselves. In line with affective events theory (AET, Weiss and Cropanzano, 1996), our work shows that targets' behavioral reactions to gossip about themselves can be understood through an emotion framework, and that predispositions shape the emotional pathways. Gossip about the self is a highly meaningful workplace event, likely to influence one's self-image (Argyle, 1969) as well as one's reputation among colleagues (Burt, 2008; Beersma and van Kleef, 2011; Feinberg et al., 2012). As such, the present results highlight that especially events that imply evaluation of the self by others, such as coworkers, arouse strong (positive and negative) emotional reactions directed at the self and at others, which inform subsequent behaviors.

Across three studies, we found that hearing positive and negative gossip aroused specific self-conscious and other-directed emotions and behavioral intentions for gossip targets. First, targets of negative (vs. positive) gossip intended to repair their mistakes, because they experienced self-conscious negative emotions (i.e., guilt and shame), especially when they scored low on core self-evaluations (CSE, Judge et al., 1997). Second, people who overheard positive (vs. negative) performance-related gossip about themselves experienced self-conscious positive emotions (e.g., pride). Third, targets of negative (vs. positive) gossip intended to retaliate against gossipers, because they experienced other-directed negative emotions (e.g., anger), especially when targets had high concerns for reputation (CR). Fourth, positive (vs. negative) gossip aroused other-directed positive emotions (e.g., liking), which increased targets' intention to affiliate with gossipers (see also Hewitt, 1972).

These findings help understand target's emotional and behavioral reactions to gossip as functional. Repair behaviors might be an adaptive response to negative gossip, helping targets avoid further deterioration of their self-views and social relationships. Intentions to retaliate against gossipers, who have harmed targets' social capital may also be functional in deterring future reputational attacks. Furthermore, positive gossip confirms valuable attributes or goal accomplishment and serves targets' fundamental need for a positive self-view (Kunda, 1990; Sedikides and Strube, 1997), potentially motivating individuals to strive for future achievements and status (Tracy et al., 2010). Moreover, positive gossip is functional in fostering a social bond between targets and gossipers, who are likely to be perceived as supportive and trustworthy allies.

Our work also clarifies that people have distinct emotional reactions to gossip and feedback about themselves, thereby indicating that the two types of self-relevant information have distinct implications for the targets' self-evaluation and reputation. Negative feedback generated higher self-conscious negative emotions and repair intentions than negative gossip, possibly because formal feedback is communicated for improvement and development purposes and increases one's sense of self-awareness and accountability. In contrast, because negative gossip is spread in one's absence (Foster, 2004) and is not clearly intended to advance performance, it may be more easily discounted by targets, thereby generating lower self-conscious negative emotions and repair intentions. Furthermore, gossip led to higher other-directed negative emotions and to lower other-directed positive emotions than feedback, suggesting that gossip is perceived as more malignant or less benign than formal feedback, possibly because it is communicated behind one's back. These results indicate that gossip is a mechanism that parallels formal communication channels in organizations and regulates group members' behavior and interpersonal relations.

In addition to the hypothesized reactions of gossip targets, the analyses revealed other effects. Consistent across Studies 1 and 3, self-conscious positive emotions predicted retaliation intentions. Positive gossip may enable targets to evaluate themselves as better than others [i.e., hubristic pride, (Tracy et al., 2010)], possibly generating retaliation intentions, because hubristic pride instigates people to establish a reputation of dominance and assert power through aggression (Tracy et al., 2010). However, other-directed positive emotions induced by positive gossip decreased retaliation intentions (Study 3) and increased repair intentions (Studies 1 and 2). Thus, positive gossip also made targets feel included, thereby motivating prosocial and reducing antisocial behaviors. As such, positive gossip generated both retaliation and affiliation intentions by arousing self-conscious and other-directed positive emotions, respectively. Furthermore, negative gossip targets were more likely to affiliate with the gossiper due to negative self-conscious (Studies 2 and 3) and other-directed emotions (Study 3). Targets who feel guilty or ashamed may see gossipers as expert observers of their shortcomings [expert power, (Kurland and Pelled, 2000)] and seek contact to obtain support or advice. In contrast, those who are angry with gossipers may seek future contact to disprove the negative gossip, or to search for retaliation opportunities.

## Theoretical Implications

Previous research highlights at least three major functions of gossip: social control, indirect aggression, and socializing (e.g., Fine and Rosnow, 1978; Dunbar, 2004; Beersma and Van Kleef, 2012). Our findings are consistent with the functional view of gossip and contribute to a broader understanding of gossip from the targets' perspective. First, negative gossip motivates targets to engage in group-benefitting behaviors and comply to norms (i.e., repair behaviors), because they may experience self-conscious negative emotions when they overhear others criticizing them for rule violations; these results help clarify (one of) the mechanisms through which gossip fosters social control in groups [see also (Sommerfeld et al., 2007; Beersma and van Kleef, 2011; Beersma and Van Kleef, 2012; Feinberg et al., 2012)]. Second, targets of negative gossip may also intend to retaliate against gossipers, due to negative other-directed emotions aroused by the negative rumors spread about themselves. These results are consistent with the view that spreading negative gossip is immoral and destructive, and generates spiraling aggression (Waddington, 2012). Third, our study showed that positive gossip targets like and want to affiliate with gossipers. While previous research indicates that gossiping facilitates formation of social bonds among gossipers (Ellwardt et al., 2012), the current results show that positive gossip can also strengthen relationships between gossipers and targets.

In line with the AET (Weiss and Cropanzano, 1996), our findings have shown that the prosocial (repair and affiliation) and antisocial (retaliation) behaviors of gossip targets are driven by affective processes, and that predispositions (CSE and CR) moderate the affective and behavioral reactions to gossip events. Future research may additionally investigate the role of other cognitive or motivational processes in shaping gossip targets' behavior, or whether gossip about the self may be experienced in a non-affective manner. Furthermore, given the differential effects of gossip vs. feedback found in Study 2, it may be interesting for the AET to distinguish between more formal vs. informal affective events at work. Our results suggest that affective reactions may differ where formal evaluations vs. gossip are concerned, and perhaps similar effects can be expected for other types of (formal vs. informal) communication at work, such as official, written communication compared to rumors.

The positive emotions generated by positive gossip are universally pleasing and can easily co-occur, as was the case in all three studies: targets were simultaneously happy with themselves and with gossipers. However, different association patterns are possible for the negative emotions aroused by negative gossip. On the one hand, gossip targets may exclusively feel negative emotions directed at the gossipers for their harmful gossiping behavior, possibly rejecting their own faults to protect their self-views (Kunda, 1990). On the other hand, targets may feel self-conscious about their shortcomings and blame gossipers for sharing the negative gossip. In Studies 1 and 2, self-conscious and other-directed negative emotions were not correlated when gossip valence was accounted for, but they were positively correlated in Study 3, suggesting that boundary conditions may apply. In Study 3 we indeed showed that the arousal of negative self-conscious and other-directed emotions depends on self-directed (CSE) and other-directed (CR) dispositional factors, respectively. Furthermore, self-conscious and other-directed negative emotions predicted whether gossip targets had prosocial (reparation) or antisocial (retaliation) intentions.

## Practical Implications

Gossip is omnipresent (Dunbar, 2004) and anyone may become a gossip target at the workplace, where gossip has consequences for the organization, groups, and individuals. As our results show, negative gossip can make targets likely to correct their shortcomings, subsequently improving group functioning. However, by complying with gossipers' informal requirements, targets may appear weak, experience reduced power or status, and face future gossip threats. Thus, gossip's normative function entails the danger that by enforcing group norms, gossipers may coerce or abuse targets. Another possibility is that negative gossip targets experience overwhelming self-conscious negative emotions and see no possibility of correcting their faults, leading to withdrawal or turnover intentions (Burt, 2008). Furthermore, targets may perceive negative gossip as unjust, and intend to harm the gossipers in return. As such, negative gossip may set in motion a spiral of aggression, transforming the workplace into a hostile environment (Grosser et al., 2012): displaying anger decreases cooperation, elicits reciprocal anger and prompts competitive and retaliatory behavior (Van Kleef and Côte, 2007).

Although gossip can have beneficial effects for groups, organizations often develop strategies to eliminate all workplace gossip (Waddington, 2012). Considering the current results, we propose that instead of banning all gossip, organizational members could become more aware of the beneficial effects of positive performance-related gossip, which strengthens targets' self-confidence and helps them build relationships with coworkers. Furthermore, managers could focus on decreasing malicious gossip, spread with the clear goal of harming others. Implementing a moral code of conduct within organizations, using less competitive incentive structures (Kniffin and Wilson, 2010), and communicating organizational goals and planned changes (Mills, 2010) are possible strategies. Furthermore, to reduce the impact of negative gossip and its consequences on antisocial behavior, organizations could give workers more opportunities to communicate evaluations regularly via feedback systems, which might reduce the transmission of gossip, or its impact on targets' emotions.

Our work may be useful in understanding how gossip influences emotions and behaviors not only for employees, but for other populations as well. Adolescents may be particularly vulnerable to gossip about themselves, because during this life stage identities are still being defined, and relationship styles are consolidated; hearing gossip about the self may be a de-stabilizing experience, that influences important life and career decisions, as well as academic motivation and success (e.g., Levy-Tossman et al., 2007; Pellerone et al., 2015). As such, repeated negative gossip experiences in adolescence may have long-term negative consequences. Our research may prove useful in predicting and preventing some of the damaging effects of gossip.

Furthermore, other groups may be especially vulnerable to negative gossip about themselves: people suffering from depression, those who are socially isolated, or people with insecure attachment styles, who are likely to have dysfunctional coping strategies, and experience extreme stress (Craparo et al., 2018). Similarly, positive gossip may benefit these groups, by enhancing positive states and adaptive behaviors, such as seeking affiliation with others. We encourage future studies to extend our research by investigating how targets from different populations respond to gossip about themselves.

## Limitations and Future Research

Our findings were consistent across different populations and using both scenario studies and critical incidents. Yet, the present study also has some limitations. First, although intentions precede behavior (Fishbein and Ajzen, 1975), we only measured behavioral intentions, and not actual behavior. To bring higher validity to our findings, future studies could investigate how emotions shape gossip targets' actual behavior.

Second, we examined a limited range of emotional and behavioral responses. Gossip may elicit other emotions and behaviors. For example, earlier research has investigated fear responses of people who hear gossip about others (Martinescu et al., 2014). The negative self-conscious and other-directed emotions we focused on in the current study may be fundamentally based on fear of losing something valuable: one's self-respect or the respect of other people. Future research should investigate other emotional reactions of gossip targets and their associated behaviors.

Third, our moderators, CSE and CR, were measured as stable dispositional factors. However, for higher validity, future research should investigate whether manipulated levels of self-evaluations and reputation concerns lead to similar results. Moreover, we only focused on moderators for negative emotions. Because the workplace is a social environment where individuals have clearly defined roles and relationships, which are likely to influence gossip targets' emotions and behaviors, other important factors should be included in future research, such as target's relationship with the gossiper, and the hierarchical positions and power levels of the gossiper, recipient, and target.

Given the differential effects of gossip and feedback on targets' emotions and behavioral intentions, it would be interesting for future research to investigate what determines whether people communicate their evaluations about targets via gossip or feedback systems. One possibility is that gossiping precedes giving feedback: first people might engage in gossip to seek and validate information (e.g., Beersma and Van Kleef, 2012), and to coordinate their actions toward gossip targets (Peters and Kashima, 2007), and afterwards approach targets with clear feedback. Furthermore, because gossip is not addressed to its targets, but may easily harm their well-being, future research should investigate whether (or when) gossipers are concerned about how gossip might affect targets, and how to increase awareness among gossipers.

## Conclusion

Our study shows that individuals who overhear gossip about themselves experience discrete emotions, defined by gossip valence and gossip's implications for self-evaluation and reputation. In turn, emotions influence targets' intentions to repair their flaws or mistakes, and to retaliate against or affiliate with the gossiper. Furthermore, target's prosocial or antisocial behaviors depend on the dispositional factors of CSE and CR which influence arousal of, respectively, self-conscious and other-directed negative emotions. In addition, this study shows that gossip and feedback have different effects on targets' emotions and behavioral intentions. Overall, our study provides core insights into emotional and behavioral responses of targets who (unintentionally) overhear gossip about themselves.

## Author Contributions

All authors contributed to the conception and design of the study. EM organized the data collection, performed the statistical analysis and wrote the first draft of the manuscript. OJ and BN wrote sections of the manuscript. All authors contributed to manuscript revision, and read and approved the submitted version.

### Conflict of Interest Statement

The authors declare that the research was conducted in the absence of any commercial or financial relationships that could be construed as a potential conflict of interest.

## Footnotes

^1^Although interpersonal liking is not strictly defined as an emotion, it is an affectively infused positive attitude toward others (see Affect Infusion Model, Forgas, 1995). As such, we use liking as a proxy for other-directed positive emotion. Our measures of liking clearly reflect the affective nature of the construct.

^2^Initially, this study had a 2 (gossip valence: positive vs. negative) x 3 (achievement goal: mastery vs. performance vs. control) design, to see if reactions to gossip are influenced by achievement goals. We collapsed the analyses across the achievement goal conditions, because no effects of achievement goals were observed.
